# Leptomeningeal metastasis from solid tumors: clinical features and its diagnostic implication

**DOI:** 10.1038/s41598-018-28662-w

**Published:** 2018-07-11

**Authors:** Zhenyu Pan, Guozi Yang, Hua He, Tingting Yuan, Yongxiang Wang, Yu Li, Weiyan Shi, Pengxiang Gao, Lihua Dong, Gang Zhao

**Affiliations:** 1 Department of Radiation-Oncology, The First Hospital of Jilin University, Changchun, 130021 China; 2 Department of Neuro-oncological Surgery, The First Hospital of Jilin University, Changchun, 130021 China; 3 Cancer Center, The First Hospital of Jilin University, Changchun, 130021 China; 4 Department of Radiology, The First Hospital of Jilin University, Changchun, 130021 China; 5 Department of Clinical Laboratory, The First Hospital of Jilin University, Changchun, 130021 China

## Abstract

In this study, we examined the characteristics and aimed to increase the knowledge of clinical features of leptomeningeal metastasis (LM). The clinical data, including initial diagnosis and treatment of primary tumor, clinical manifestations, neuroimaging findings, cerebrospinal fluid (CSF) examination, were analyzed. For the patients with adenocarcinoma/breast cancer, the incidence of cranial lesions and cranial nerve paralysis was obviously higher than patients with small cell lung cancer. Whereas, the incidence of involvement of intravertebral canal was obviously lower than that of small cell lung cancer. Patients with adenocarcinoma/breast cancer showed more incidence of leptomeningeal enhancement compared to those with small cell lung cancer. Persistent severe headache was noticed in those with squamous carcinoma, and usually showed absence of abnormally LM-related neuroimaging and CSF cytological findings, which resulted in a challenge in the diagnosis of LM from squamous carcinoma. Patients with different primary tumors showed differential clinical features. Significant differences were observed in clinical features between patients with adenocarcinoma/breast cancer and small cell lung cancer. Our study contributes to the understanding of clinical characteristics of LM, and contributes to improvement of LM diagnosis in clinical practice.

## Introduction

Leptomeningeal metastasis (LM), a lethal complication of malignant tumors, refers to involvement of the cerebrospinal fluid (CSF) and leptomeninges by tumor cells. Nowadays, it is considered the third most common metastatic complication involving the central nervous system (CNS)^1^. The LM-related clinical manifestations and neuroimaging findings were varieties as the involvement of whole central neuraxis. Up to now, there is no ideal golden standard for LM diagnosis due to lacking of non-invasive diagnostic methods with high specificity and sensitivity. The diagnosis of LM is mainly depending on detection of malignant cells in CSF by cytological examination, radiographic manifestations as revealed by neuraxis imaging, and other accessory clinical findings. There is a need to raise awareness of the clinical features.

Identification of cancer cells in CSF using cytological examination is currently considered as the gold standard test for LM diagnosis. Nevertheless, false negativity still exists despite multiple examinations^2^. Besides that, as an invasive examination, CSF cytology is not proposed as a common examination.

With improved visualization of the subarachnoid space and improvement of sensitivity by high field magnetic resonance imaging (MRI), imaging has become the initial, even sole, diagnostic tool in the MRI era^3,4^. In the past decade, MRI has been regarded as an important component of LM diagnosis, and the sensitivity of MRI varied from 20% to 91%^5^. In recent years, neuro-imaging is the most common auxiliary examination utilized for identifying patients with clinical suspicion of LM and being adequate to establish the LM diagnosis with a typical clinical presentation^5^. However, the neuroimaging findings of LM are diverse, complex and usually nonspecific^5^. Its lower specificity precludes it from replacing cytology as the gold standard test for diagnosis^5^.

In recent years, circulating tumor cells or circulating tumor DNA (ctDNA) has been utilized in the CSF related examinations for LM from solid tumor. Such technique has been usually used in the basic research of LM^6–8^. In some clinical studies, it showed that rare cell capture technology could be used to detect circulating tumor cells in the CSF of patients with LM from epithelial tumors^9,10^. However, such method is not used commonly. With the advances of technology, these methods may be an optimal examination option with high sensitivity and specificity for CSF test, which improves the diagnostic efficiency of LM.

Despite great strides have been made, the diagnosis of LM from solid tumors is still in a dilemma^4,11^. The improvement of LM diagnosis depends on enhancement of appreciation and identification for clinical features. In this study, we undertook a retrospective study on clinical data from solid tumor patients with LM over 6 years. We aimed to increase the knowledge of clinical features of LM, which may contribute to the identification of LM patients and improvement of the clinical diagnosis.

## Results

### Patient characteristics

Over past 6 years, 205 solid tumors patients diagnosed with LM were recorded in LM database of our hospital. In total, 42 cases were excluded, including 28 patients with primary CNS tumors, 8 with lymphoma and 6 with unknown malignancy. A total of 163 patients were enrolled in this study, and the general information of the patients was shown in Table 1. LM was the initial manifestation of cancer in 19 patients. For the remaining 144 patients, 28 (19%) were diagnosed with LM during the initial treatment course of primary tumor, while other 116 (81%) were diagnosed with recurrence. Among the 84 patients with pulmonary adenocarcinoma, 21 (25%) showed LM at 2–18 months (median, 12 months) after the tyrosine kinase inhibitor (TKI) target therapy, including gefitinib (n = 14), erlotinib (n = 3), icotinib (n = 2), or gefitinib combined with erlotinib (n = 2). Twenty-two (13%) had a history of brain metastasis, among which 18 (11%) had received surgery and/or brain radiotherapy previously.

**Table 1:** General information of the patients at initial LM diagnosis.

Characteristic	N (%)
**Gender**	
Male	77 (47%)
Female	86 (53%)
**Median age**	57 yrs (range 21–76 yrs)
**Pathological features of the primary disease**	
Pulmonary adenocarcinoma	84 (52%)
Pulmonary adenosquamous carcinoma	3 (2%)
Squamous cell lung cancer	7 (4%)
SCLC	29 (18%)
Breast cancer	26 (16%)
Gastric adenocarcinoma	7 (4%)
Others*	5 (3%)
**Median KPS**	40 (range 10–100)
**GCS**	
15	87 (53%)
13~14	53 (33%)
9~12	23 (14%)
**Median time interval between diagnosis of primary tumors and LM**	13 months (range 0–109 months)
**LM as initial manifestation of cancer**	19 (12%)
Pulmonary adenocarcinoma	14 (74%)
Gastric adenocarcinoma	2 (11%)
SCLC	2 (11%)
Primary intracranial melanoma	1 (5%)
**Systemic disease status**	
Stable	59 (36%)
Free	17 (10%)
Active	77 (47%)
Unevaluated	10 (6%)

SCLC, small cell lung cancer; KPS, Karnofsky performance score; GCS, Glasgow Coma Scale.

*Including double primary cancers of breast cancer and lung cancer (n = 1), hepatocellular carcinoma (n = 1), laryngeal squamous cell carcinoma (n = 1), rhabdomyosarcoma of paranasal sinus and nasal cavity (n = 1), primary intracranial melanoma (n = 1).

### Establishing the diagnosis of LM

According to the diagnosis criteria, the number of patients diagnosed by each item was 131, 10, 67, 114 and 18, respectively.

### Clinical manifestations

The majority of the patients showed poor prognostic factors and pleomorphic neurological symptoms/signs at the initial diagnosis of LM. Only 4 patients (2%) with pulmonary adenocarcinoma were asymptomatic. They received MRI examination as a screening or a review for brain metastasis. The neuroimaging findings showed leptomeningeal abnormal enhancement. Further CSF cytological examination validated the LM diagnosis. Almost of the patients (98%) showed neurological symptoms and signs in three domains at the LM diagnosis (i.e. cerebral hemisphere, cranial nerve, and the existing nerve roots).

**Figure 1:**
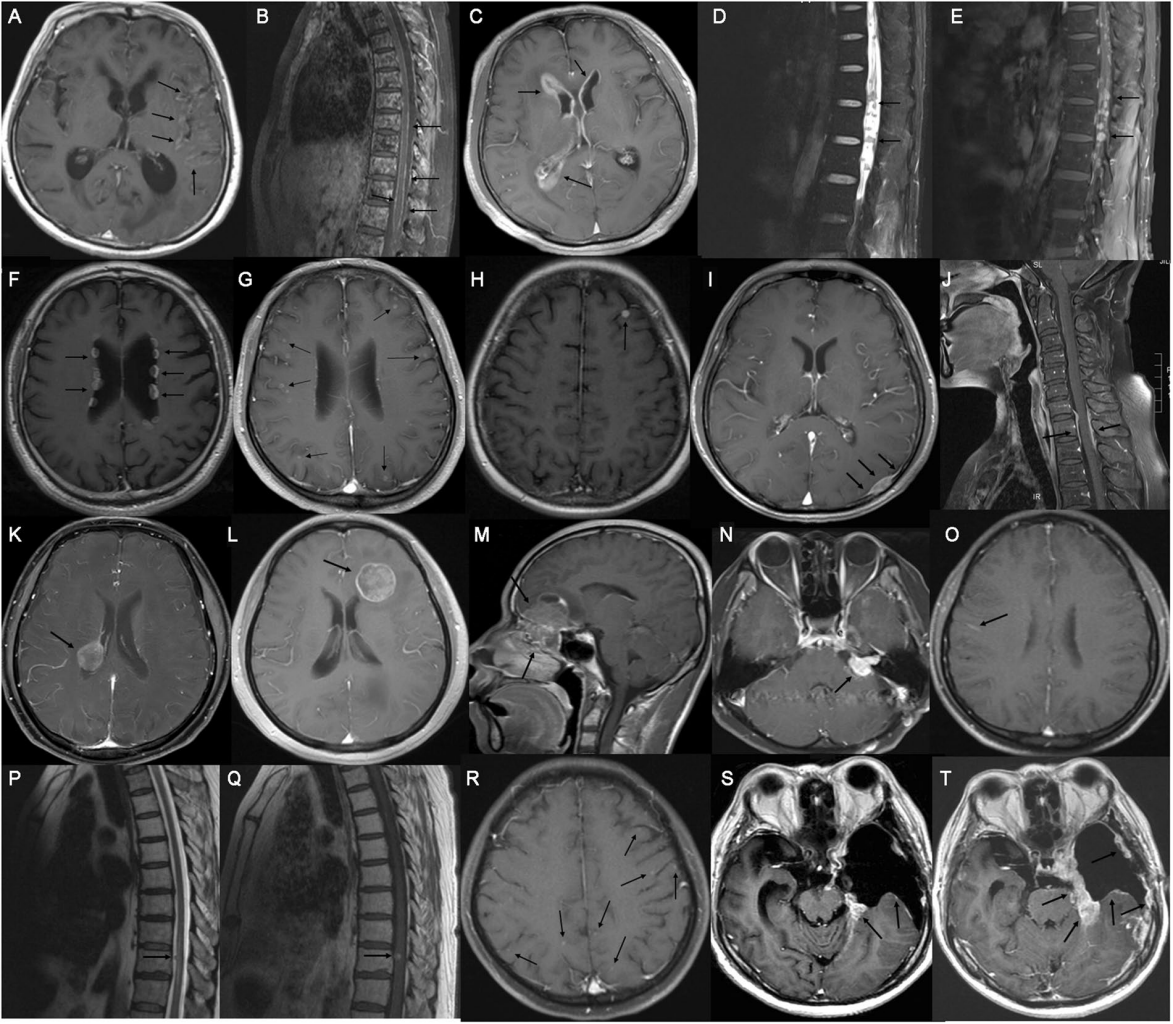
Neuroimaging findings. (**A**) Diffuse pial enhancement in brain sulcus, especially the left temporal lobe. (**B**) Diffuse leptomeningeal enhancement in vertebral canal. (**C**) Enhancement of subependyma in the bilateral ventricles. (**D**) Multiple nodular lesions in spinal canal in T2-weighted sequences. (**E**) Obvious enhancement in contrast enhanced T1-weighted sequences. (**F**) Multiple nodular lesions with enhancement in the wall of lateral ventricles. (**G**) Enhancement of metastatic nodules in cerebral cortex. (**H**) Metastatic lesion in subarachnoid space. (**I**) Dural enhancement in left occiput and rat-tail sign were observed. (**J**) Dural enhancement in local spinal canal. (**K**) Metastatic lesion in the posterior horn of the right lateral ventricle. (**L**) Metastatic lesion correlated to the anterior horn of the left lateral ventricle. (**M**) The rhabdomyosarcoma of paranasal sinus and nasal cavity invaded across the bottom of anterior cranial fossa and the frontal lobe. (**N**) The enhancement of auditory and facial nerves at the left cerebellopontine angle. (**O**) Fuzzy pial enhancement presented at the sulcus and gyrus. (**P**,**Q**) A Lesion of implantation metastasis on the surface of spinal cord. (**R**) The occult metastatic nodules in cerebral cortex. (**S**) Subdural hydroma at the left tempus. Slight enhancement of the local dura mater. (**T**) One month later, it showed worse subdural effusion and diffusely dural thickening and enhancement.

Most of the patients suffered from neurologic symptoms prior to LM diagnosis, including dizzy, cervical discomfort, or mildly cranial nerve paralysis such as tinnitus or diplopia that was usually neglected. Generally, those symptoms were deteriorative in a few days or weeks. At LM diagnosis, headache was the most common symptom (82%). A few patients (41%) presented nausea and vomiting after deterioration of headache accompanied with rapid weight loss in a short-term. Seventy-two patients (44%) showed mental obtundation, speech disorder and hypersomnia. Mental disorders were observed in 22 patients (13%) mainly manifested as personality change, restless or euphoria. Eleven patients (7%) showed encephalopathy characterized by dullness, hypersomnia, memory loss, decrease of response and cognition, as well as speech disorder. In addition, 27 patients (17%) suffered from seizure. The incidence of seizure was obviously higher than the previous report^5^, which was partly because of most patients enrolled in this study with late-stage LM. Ninety-two patients showed cranial nerve paralysis mainly presenting as tinnitus and hearing loss (40%), diplopia (37%), blurred vision (18%), facial numbness (9%), and dysphagia (6%). Seventy-one patients (44%) showed multiple cranial nerve paralysis. The incidence of spinal nerves symptoms (15%, 24/163) was lower than those of brain (88%) and cranial nerves (56%), which were mainly manifested as disorders in movement and sensation (13%), radiculalgia (8%), and abnormalities in the urination and defecation (9%). One patient with cervical and thoracic spinal involvement (Fig. 1J) showed amyotrophy of hands and distal end of upper limb within 6 months prior to LM diagnosis.

In this study, three patients presented rare manifestations, including central fever (n = 1) and diabetes insipidus (n = 2) which had been never reported in previous literatures. The central fever was manifested as persistent high fever, and infectious disease was excluded after CSF and serum examinations. The patient was conscious, and was finally died within a few days. Among the 2 patients with diabetes insipidus, 1 patient with lung adenocarcinoma suffered from the diabetes insipidus upon initial LM diagnosis, while the other 1 with breast adenocarcinoma showed such condition during LM recurrence 2 years later after initial LM diagnosis and treatment. The diabetes insipidus lasted for 1.7 and 2.7 months respectively, and was alleviated completely after effective LM-related treatment.

Three breast cancer patients with long-term focal peripheral nervous involvement including cervical and thoracic spinal meninges (n = 1) and cranial nerves (n = 2) were diagnosed with LM by CSF cytology. Two patients received both involved-field radiotherapy and intrathecal chemotherapy. One patient died from disease progression with a survival time of 18.5 months, while another patient was still alive with a survival time of more than 27 months. The third patient merely received involved-field radiotherapy, and died from systemic disease progression with a survival time of 13 months.

Neurological remission was generally achieved a few days later after effective therapy. As the disease progress and recurrence, the previous symptoms presented deterioration or relapse. Nineteen patients presented spinal nerve symptoms as a new symptom 1–8 months later after initial LM diagnosis, including14 patients with adenocarcinoma and breast cancer and 5 patients with small cell lung cancer (SCLC). Additionally, fourteen patients suffered a seizure during effective intrathecal chemotherapy, especially after the first cycle of intrathecal chemotherapy. It was probably due to some stimulus factors releasing into CSF caused by damage of tumor cells and abnormal discharge. Nevertheless, seizure also occurred with the progression of LM disease.

### Neuroimaging findings

In total, 156 patients received neuroimaging examination including contrast MRI or CT scan, among which 147 patients received MRI examination and 9 patients unable to undergo MRI received contrast cranial CT scan. The MR sequences were T1-weighted images without contrast, fat suppression T2-weighted, fluid attenuation inversion recovery (FLAIR) sequences and T1-weighted sequences with contrast. One hundred and forty-four patients received MRI examinations on brain, while sixty-eight patients received spinal MRI, including lumbar spine (n = 66), thoracic spine (n = 23) and cervical spine (n = 10).

Sixteen patients (10%) showed normal neuroimaging. The majority of patients (90%) showed neuroimaging features of LM **(**Table 2**)**, which were divided into two groups according to the previous literatures^12,13^: (i) diagnostic features (43%), including leptomeningeal enhancement in brain **(**Fig. 1A**)** or vertebral canal **(**Fig. 1B**)**, subependymal enhancement **(**Fig. 1C**)**, multiple nodules of implantation metastases in vertebral canal **(**Fig. 1D,E**)** and ventricles **(**Fig. 1F**)**; (ii) suggestive features (73%), including nodular enhancement in cerebral cortex **(**Fig. 1G**)**, metastatic lesion approaching sulcus and gyrus (Fig. 1H**)**, dural enhancement in intracalvarium **(**Fig. 1I**)** or vertebral canal **(**Fig. 1J**)**, bulky metastasis inside or proximity to ventricles **(**Fig. 1K,L**)**, direct invasion to intracalvarium by head and neck malignancy **(**Fig. 1M**)**, cranial nerve enhancement **(**Fig. 1N**)**, or communicating hydrocephalus. Forty-four patients (28%) showed suggestive and diagnostic imaging features simultaneously.

**Table 2:** Clinical features of patients with various pathological types at the initial LM diagnosis.

Clinical features	Lung adenocarcinoma^&^ (n = 87)	Breast cancer (n = 26)	SCLC (n = 29)	Squamous cell carcinoma (n = 8)^*^	Large cell Lung cancer (n = 2)	Gastric adenocarcinoma (n = 7)	Double primary cancers^#^ (n = 1)	Hepatocellular carcinoma (n = 1)	CNS Malignant melanoma (n = 1)	Head and neck Rhabdomyosarcoma (n = 1)	Total (n = 163)
**Clinical manifestations**	87	26	29	8	2	7	1	1	1	1	163
**Symptoms in brain**	82	24	16	8	2	7	1	1	1	1	143(88%)
Headache	78	22	12	8	2	7	1	1	1	1	133(82%)
Vomiting	34	12	8	4	1	4	1	1	1	1	67(41%)
Mental obtundation and slurred speech	47	12	3	2	1	5	1	0	0	1	72(44%)
Mental disorder	10	3	9	0	0	0	0	0	0	0	22(13%)
Encephalopathy	6	1	2	0	1	1	0	0	0	0	11(7%)
Neck discomforts/rigidity	23	10	8	2	1	5	1	1	1	1	53(33%)
Seizure	10	5	4	1	1	4	1	0	1	0	27(17%)
**Symptoms in cranial nerves**	55	14	10	1	2	6	1	1	1	1	92(56%)
Diplopia	32	12	6	0	1	5	1	1	1	1	60(37%)
Tinnitus and decrease of audition	39	8	7	1	2	6	1	1	1	0	66(40%)
Blurred vision	19	7	2	0	1	0	0	0	0	1	30(18%)
Facial numbness	5	3	2	0	0	2	1	1	0	0	14(9%)
Bucking and dysphagia	2	1	3	0	1	1	0	1	0	0	9(6%)
**Symptoms in spinal nerves**	6	4	10	1	2	1	0	0	0	0	24(15%)
Dyskinesia and sensory changes	6	4	8	1	2	1	0	0	0	0	22(13%)
Radiculalgia	3	2	5	1	1	0	0	0	0	0	12(7%)
Urination and defecation disorder	3	2	6	1	2	0	0	0	0	0	13(8%)
**Neuroimaging examination**	81	25	29	8	2	7	1	1	1	1	156
**Diagnostic features**	32	11	15	1	2	4	1	0	1	0	67(43%)
Leptomeningeal enhancement in brain	29	6	1	1	1	3	1	0	1	0	42(27%)
Leptomeningeal enhancement in vertebral canal	3	3	4	0	2	1	0	0	0	0	13(8%)
Implantation metastasis in vertebral canal	3	1	6	0	2	0	0	0	0	0	12(8%)
Subependymal enhancement	4	1	7	1	0	0	0	0	0	0	13(8%)
Implantation metastasis in ventricles	4	1	6	1	1	0	0	0	0	0	13(10%)
**Suggestive features**	68	19	17	4	2	1	0	1	1	1	114(73%)
Dural enhancement	7	4	1	0	0	0	0	0	0	1	13(8%)
Metastatic lesions approaching subarachnoid space	49	15	8	1	2	0	0	1	1	1	78(50%)
Metastatic lesions approaching ventricles	5	1	9	2	0	0	0	1	0	0	18(12%)
Enhancement of cranial nerves	1	2	0	0	0	0	0	1	0	0	4(3%)
Communicating hydrocephalus	6	1	0	2	0	1	0	0	0	0	10(6%)
**Negative**	8	2	1	3	0	2	0	0	0	0	16(10%)
**CSF examination**	84	26	27	8	2	7	1	1	1	1	158
Positive cytology	80	26	19	3	2	7	1	1	1	1	141(89%)
Aberrant biochemical analysis	72	25	26	3	2	7	1	1	1	1	139(88%)

SCLC, small cell lung cancer; CSF, cerebrospinal fluid; As the morphology of the cancer cells in CSF was in line with adenocarcinoma in three pulmonary adenosquamous carcinoma patients, and the clinical manifestations were consistent with the patients with pulmonary adenocarcinoma. These patients were categorized into the adenocarcinoma group.

^**&**^Including pulmonary adenocarcinoma (n = 84), pulmonary adenosquamous carcinoma (n = 3); ^**#**^double primary cancers of breast cancer and lung cancer; ^*^including squamous lung carcinoma (n = 7), laryngeal squamous cell carcinoma (n = 1).

Leptomeningeal enhancement was the most frequent diagnostic imaging feature which was mostly located at sulcus and gyrus of cerebrum or cerebellum. It was worthy to note that some enhancement was fuzziness **(**Fig. 1O**)**. Also, it was necessary to refer to the surrounding or contralateral structure to distinguish from normal enhanced vessels. Occasionally, a lesion of implantation metastasis was observed on the surface of spinal cord **(**Fig. 1P,Q**)**. Ventricular implantation was a diagnostic feature characterized by multiple disseminated lesions in ventricles **(**Fig. 1F**)**. Additionally, lesion invaded ventricle was a suggestive feature, which was manifested as a lesion approaching or involving ventricle **(**Fig. 1K,L**)**. The most common suggestive imaging finding was lesions approaching subarachnoid space with an incidence of 54% in this study. Meanwhile, some metastatic nodules were obscure **(**Fig. 1R**)**. Besides, we also observed a rare neuroimaging finding. One patient showed severe subdural hydroma after surgery for the management of metastatic lesion **(**Fig. 1S**)**, which may be correlated to adhesion of focal arachnoid mater and valve formation. One month later, irregular thickening and diffuse enhancement of dura mater were observed **(**Fig. 1T**)**.

Most of the neuroimaging examinations were performed to the sites of neurological symptoms in this study. Nevertheless, 4 patients with small cell carcinoma received spinal MRI as a screening showing involvement of vertebral canal, accompanied with no related symptoms/signs in spinal nerves. In addition, eleven patients with adenocarcinoma, three with breast cancer and five with small cell carcinoma presented symptoms of spinal nerves 1–8 months later after initial LM diagnosis, and then the neuroimaging examinations detected leptomeningeal enhancement in vertebral canal (n = 8) and implantation metastasis in vertebral canal (n = 11).

As the neuroimaging features of LM commonly are not measurable at least as defined by current response criteria, the one-dimensional response evaluation criteria in solid tumor (RECIST) are not appropriate for evaluation of LM. Therefore, the neuroimaging examination was not proposed to every patient as a review. A total of 62 patients received cranial MRI/CT 1–24 months later after concomitant therapy, 39 showed leukoencephalopathy. All the 24 patients undergoing MRI scan over 6 months later after therapy were confirmed with leukoencephalopathy.

### CSF examination

A total of 158 patients received CSF examination. CSF samples were collected by lumbar puncture (1–6 tests per patient). Based on our previous study^14^, CSF cytological examination was carried out using Thinprep plus papanicolaou stain method. At least 7 mL CSF sample was collected for cytological examination. Sample was promptly subjected to examination after collection (less than 40 min). About 1–2 mL CSF was used in conventional biochemical test. Abnormal result was defined as white blood cell of 4/mm^3^, protein of >0.45 g/L, and glucose of <2.3 mmol/L.

Intracranial hypertension (>200 mmH_2_O) was observed in 114 patients (70%). One hundred and thirty-nine (88%) patients showed abnormal results of CSF biochemical examination, including elevation of white blood cells (52%), CSF protein (78%), and decrease of glucose (41%, Table 2**)**.

**Figure 2:**
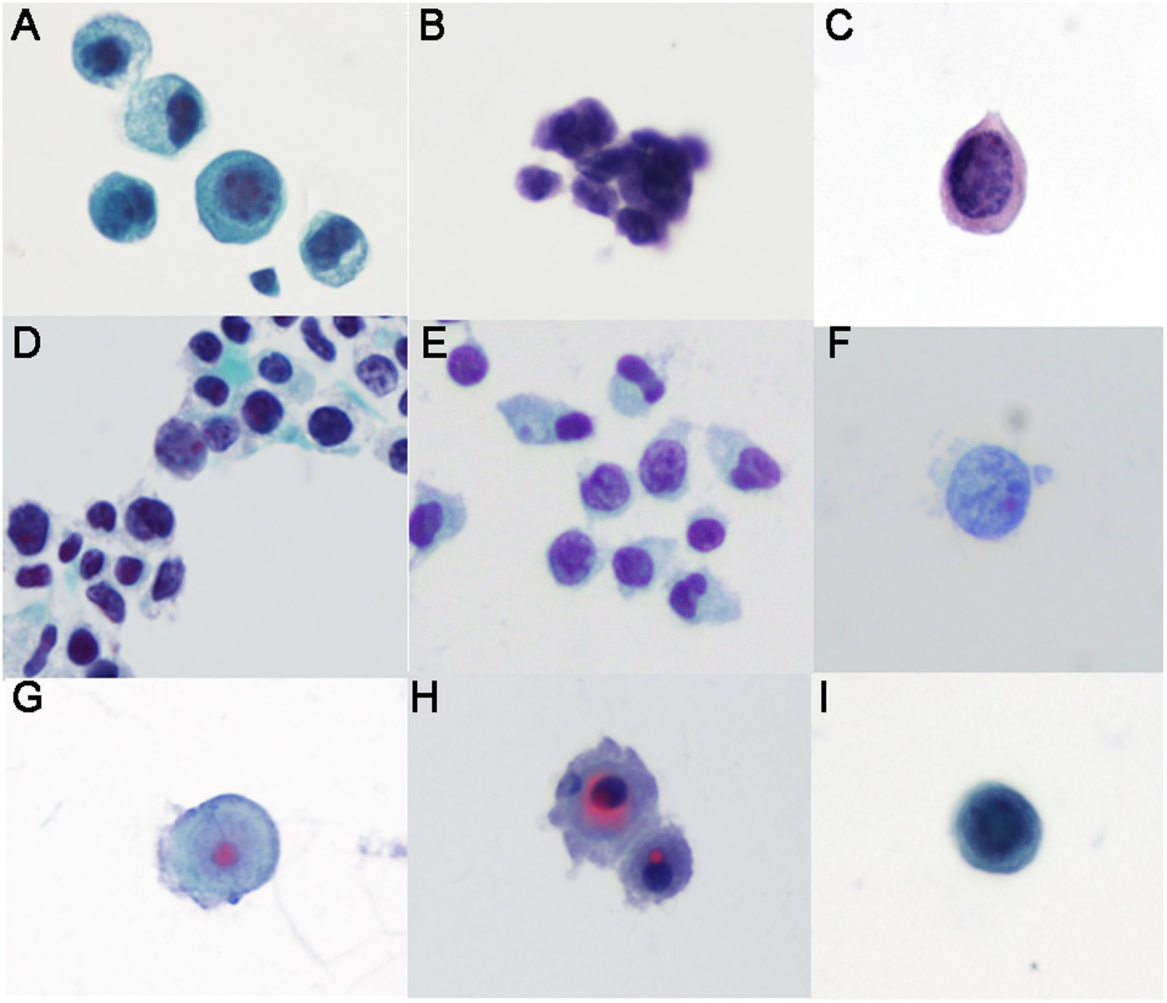
Malignant cells in CSF. (**A**) Adenocarcinoma: scattered or clustered distribution; intensely stained cytoplasm with occasionally observed vacuoles; most cells had intensely and unevenly stained chromatin and some showed fine granules; most cells had an irregular number of nucleoli, which were irregular in size and stained deep-red. (**B**) Small-cell lung cancer: mostly clustered; smaller than other types of tumor cells; greatly increased nucleo-cytoplasmic ratio, nuclei appearing as naked nuclei; single nucleus in most cells; intensely stained coarse granular chromatin; nucleoli not clearly visible. (**C**) Squamous cell carcinoma: scattered distribution; intensely stained cytoplasm; pink stained keratinized cytoplasm in some cells; intensely stained, flocculated or granular chromatin, some multi-nucleated cells; small obscure red-staining nucleoli in most cells located toward the edge of nucleus. (**D**) Middle differentiation Squamous cell carcinoma: clustered distribution or scattered; increased pleomorphism, smaller than Adenocarcinoma and Squamous cell carcinoma; greatly increased nucleo-cytoplasmic ratio, nuclei appearing as naked nuclei; intensely stained coarse granular chromatin; red-staining nucleoli in some cells with smaller size; some cells multi-nucleated. (**E**) Low differentiation Squamous cell carcinoma: similar to middle differentiation Squamous cell carcinoma; nucleoli not clearly visible. (**F**) Large-cell lung cancer: scattered distribution; boundary not clearly defined; single nucleus in most cells; pale stained cytoplasm with large nuclear-cytoplasimc ratio, nuclei appearing as naked nuclei; intensely stained coarse granular chromatin; obscure red-staining nucleoli in some cells with smaller size. (**G**) Hepatocellular carcinoma: scattered distribution, regular shape; clearly defined boundary; intensely stained cytoplasm; intensely and uniformly stained chromatin; a single large, round, and bright red-staining nucleolus found in each of the center of nuclei; some cells multi-nucleated. (**H**) Malignant melanoma: scattered or clustered distribution; pseudopodia-like membrane protrusions at the cell periphery; intensely stained nuclei and cytoplasm; some cells multi- nucleolus; red-staining nucleolus with irregular size found in the nucleus; some cells multi-nucleated; presence of black granular substances in irregular size in the nucleus or cytoplasm. (**I**) Rhabdomyosarcoma: scattered distribution; increased pleomorphism, intensely stained nuclei and cytoplasm; greatly increased nucleo-cytoplasmic ratio; red-staining nucleolus in partial cells located toward the edge of nucleus.

The initial detection rate of CSF cytology was 78% (124/158). The total positive rate was 89% (141/158). Malignant cells and suspicious malignant cells were detected in 131 and 10 patients, respectively **(**Table 2**)**. For the various cytopathological types of primary tumors, the CSF cancer cells presented differential morphologies **(**Fig. 2**)**. Several malignant morphological features were used for the judgment of cancer cells^15^. However, despite receiving two or more times CSF examinations, aberrant cells presenting insufficient malignant characteristics were detected in 10 patients (6%). Those were finally defined as suspicious malignant cells.

Seventy-four patients received CSF cytology as a review after LM-related treatment, including pulmonary adenocarcinoma (n = 34), pulmonary adenosquamous carcinoma (n = 3), small cell lung cancer (n = 13), squamous lung carcinoma (n = 2), large cell lung cancer (n = 2), gastric adenocarcinoma (n = 4), breast cancer (n = 12), hepatocellular carcinoma (n = 1), laryngeal squamous cell carcinoma (n = 1), rhabdomyosarcoma of paranasal sinus and nasal cavity (n = 1) and primary CNS melanoma (n = 1). Twenty-three (31%, 23/74) patients presented CSF cytological clearance including 3 with adenocarcinoma, 11 with small cell carcinoma, 1 with squamous cell carcinoma, 2 with large cell carcinoma, 3 with breast cancer, 1 with hepatocellular carcinoma, 1 with laryngeal squamous cell carcinoma and 1 with rhabdomyosarcoma. The CSF cytological clearance rate was extremely low (7%) in adenocarcinoma. Additionally, our previous study had revealed that CSF cytological clearance showed no correlation with either clinical response or survival time^16^.

### Contrast of clinical features in different pathological types

Patients with different types of primary tumors showed differential clinical manifestations at initial diagnosis of LM **(**Table 2**)**. The adenocarcinoma and breast cancer patients showed similar clinical features (Supporting Information 1). We further analyzed the difference of clinical features in patients with adenocarcinoma/breast cancer (n = 121) and SCLC (n = 29, Supporting Information 1). Most of the adenocarcinoma and breast cancer patients (94%, 114/121) were diagnosed with LM at the onset of brain or cranial nerve symptoms/signs, which was significantly higher than SCLC (P < 0.001). The incidence of headache in patients with adenocarcinoma and breast cancer was remarkably higher than SCLC patients [95% (108/114) vs. 41% (12/29), P < 0.001]. Severity of headache in most of the adenocarcinoma patients seemed to be worse than SCLC. In addition, 72 patients presented mental obtundation and slurred speech, among which 65 (90%) were adenocarcinoma and breast cancer and was significantly higher than SCLC (P < 0.001). Most of these patients (62/65, 95%) accompanied with severe headache and/or vomiting. The incidence of cranial nerves symptoms in adenocarcinoma and breast cancer patients was significantly higher than in SCLC patients [63% (76/121) vs. 34% (10/29), P = 0.006], such as diplopia (P = 0.039) and tinnitus and decrease of audition (P = 0.044). Furthermore, the incidence of spinal nerves symptoms in adenocarcinoma and breast cancer patients (9%, 11/121) was significantly lower than in SCLC patients [9% (11/121) vs. 34% (10/29), P < 0.001], including dyskinesia and sensory changes (P = 0.02), urination and defecation disorder (P < 0.001) and radiculalgia (P = 0.005). The incidence of mental disorders in SCLC was higher than in adenocarcinoma and breast cancer [31%, (9/29) vs. 11% (13/121), P = 0.013]. Additionally, all of the squamous cell carcinoma patients (n = 8) showed severe and steady headache, merely 2 (25%) showed mental abnormality or consciousness disorder, and only 1 (13%) presented spinal nerves symptoms.

The positive rate of diagnostic imaging features in SCLC was higher than adenocarcinoma and breast cancer [52% (15/29) vs. 42% (48/114), P = 0.245]. The positive rate of diagnostic imaging features was the lowest in squamous carcinoma patients (13%, 1/8). The positive rate of suggestive imaging features was 77% (88/114) in adenocarcinoma and breast cancer patients significantly higher than in SCLC patients (59%, 17/29, P = 0.043). For neuroimaging findings, statistical difference was noticed in the adenocarcinoma patients compared to that of SCLC, including leptomeningeal enhancement (P = 0.004), implantation metastases in vertebral canal (P < 0.001), subependymal enhancement (P = 0.002), implantation metastases in ventricles (P < 0.001), metastatic lesion approaching subarachnoid space (P = 0.002) and metastatic lesions approaching ventricles (P < 0.001). The positive rate of CSF cytology in adenocarcinoma and breast cancer patients was significantly higher than in SCLC patients [97% (114/118) vs. 70% (19/27), P < 0.001]. The positive rate of CSF cytology was lowest in squamous cell carcinoma patients (34%, 3/8).

### Treatment and follow-up

Ninety-one patients (56%) received treatment after LM diagnosis, including intrathecal chemotherapy (n = 80), involved-field radiation therapy (n = 71), systemic chemotherapy (n = 44), and epidermal growth factor receptor tyrosine kinase inhibitor (EGFR-TKI) therapy (n = 11). Seventy-six patients were followed up until death, and 13 patients were still survival in the follow up. Others were lost in follow up.

## Discussion

In this retrospective study, we reviewed a cohort of solid tumor patients diagnosed with LM in our hospital over the past 6 years, in order to investigate the clinical features. Unlike the previous reports, the majority of the patients showed poor prognostic factors and pleomorphic neurological symptoms/signs at initial LM diagnosis, and the median KPS score was 40. The study revealed the differential clinical features among diverse primary tumors for the first time. Moreover, a few rarely LM-related complications were observed, which had been never reported before, such as central fever, diabetes insipidus, and myatrophy. Our study benefits for enhancing awareness of clinical features of LM.

Most of previous studies about LM clinical features were focused on the analysis of clinical data obtained from LM patients with single or multiple tumor types^3,4,12,13,17–19^, however, few studies focused on the contrast between differential primary tumors or pathological types. We posted a contrast between different primary tumors. Our data indicated that pulmonary adenocarcinoma and breast cancer patients showed similar clinical features. However, compared with adenocarcinoma/breast cancer, significant differences of clinical features were observed in LM patients with SCLC. Despite unknown mechanism of such features, our study is beneficial to increase the knowledge of clinical features of LM.

EGFR-TKI therapy has been approved to be effective for treatment of LM from pulmonary adenocarcinoma^20,21^. In this study, LM occurred in 21 patients had received effective TKI therapy previously. Among these patients, 14 showed systemic disease free or stable, and the other 7 showed progressive systemic disease at LM diagnosis. This suggested that TKI therapy presents diverse effects on cancer cells in CSF and extra-CNS.

The spread of cancer cells into CSF could be confirmed by diagnostic neuroimaging features such as leptomeningeal pathological enhancement and multiple implantation metastases in ventricles or vertebral canal^5,13,17^. However, it is important to note that any irritation of the leptomeninges (e.g. subarachnoid blood, infection and inflammation) can result in enhancement on MRI. We had ever noticed a breast cancer patient presenting headache and other LM-related manifestation. Cranial MRI showed diffuse leptomeningeal enhancement. CSF cytology was negative. Ahead of those clinical manifestations, the patient had been showing cerebrospinal rhinorrhea due to osseous metastasis of skull base for 2 months. The symptoms were attenuated after administration of antibiotics. Thus, the LM diagnosis was excluded. Meanwhile, another breast cancer patient presented multiple enhancements in brain, and was finally diagnosed with multiple sclerosis.

There are some potential differential diagnoses of neoplastic meningitis, such as bacterial, viral, fungal or autoimmune meningitis. Patients with infectious meningitis, such as bacterial and viral or fungal meningitis, usually show a past history of infectious disease. Meanwhile, these patients commonly present fever and other systemic symptoms. The presentation of LM differs from that of bacterial or hemorrhagic meningitis, as fever, photophobia and meningismus are extremely uncommon^5^. Additionally, compare with LM, the infectious meningitis usually show a rapid onset, with more severe meningeal irritation. Autoimmune meningitis can occur at any stage of autoimmune disease and can be a main symptom, as a neurological complication particularly of systemic autoimmune diseases such as systemic lupus erythematosus and Behçet disease. In meningitis associated with autoimmune disease, the main symptoms are fever, headache, nausea, and neck stiffness, which are similar to those with infectious or malignant conditions^22^. A malignant history or autoimmune disease history is the basis for diagnosis. Finally, CSF cytology is required for the diagnosis. To be exact, identification of cancer cells in CSF is the most important basis for LM diagnosis. Consequently, the relevant differential diagnosis should be excluded firstly if cancer cell was not detected in CSF.

Lesions showing suggestive imaging features could indicate occurring of LM, such as nodular enhancement in cerebral cortex, metastatic lesion approaching subarachnoid space (sulcus/gyrus of brain, cerebral cortex metastasis and intraventricle metastasis), dural enhancement in intra-calvarium and vertebral canal, bulky metastasis inside or proximity to ventricles, approach to the subarachnoid space or ventricles. Leptomeninges can be involved directly by these lesions, and LM would occur in case of cancer cells entering subarachnoid space and proliferating in CSF. Thus, these imaging findings suggest a possibility of LM. On this basis, CSF cytology is obligatory for the establishment of diagnosis. In this study, 43% patients showed diagnostic imaging findings, whereas 73% showed suggestive imaging findings. As above mentioned, in most cases, the identification of malignant cell by CSF cytology was still the key feature validating LM. Even though a patient presented diagnostic neuroimaging feature, the final diagnosis should be combined with malignant history, clinical manifestations and other accessory information.

Entire neuraxis MRI is proposed for LM patients. However, it is not available in every hospital due to limited medical condition. In this study, patients with SCLC show higher incidence of involvement of vertebral canal which may be present asymptomatic especially for the lumbar and sacral segment. We propose the central axis MRI examination especially for lumbosacral segment as a common screening for LM patients with SCLC.

About 10% of the patients showed negative neuroimaging in this study, among which 56% (9/16) showed a Karnofsky pereformance status (KPS) scale of 20–40 and the intracranial pressure of more than 350 mmH_2_O. Meanwhile, some patients showed diagnostic neuroimaging findings such as leptomeningeal enhancement, and the symptoms were mild or moderate. These indicated that neuroimaging finding can not reflect LM patients’ conditions.

In this study, compared with adenocarcinoma/breast cancer patients, SCLC patients showed significant differences in clinical features. Patients with adenocarcinoma and breast cancer were more susceptible to cranial nerves palsy and headache, mental obtundation and slurred speech, while those with SCLC showed higher incidence in spinal nerves and mental disorder. Patients with adenocarcinoma and breast cancer showed significantly higher positive rates of CSF cytology compared to those with SCLC. Thus, CSF cytology is an effective method for the diagnosis of LM originated from adenocarcinoma and breast cancer. The cytological positivity for SCLC was lower than adenocarcinoma and breast cancer, however, the neuroimaging sensitivity for SCLC was superior to adenocarcinoma and breast cancer. Moreover, in line with previous report, it was difficult to detect cancer cells in CSF for LM patients with squamous cell carcinoma^23^. Meanwhile, the imaging sensitivity of LM patients with squamous cell carcinoma was merely 62.5% (5/8). It is still a challenge for the diagnosis of LM from squamous cell carcinoma.

The diagnosis of LM is still in a dilemma due to absence of method with high specificity and sensitivity. In clinical practices, a group of LM patients (approximately 25–30%) present nonspecific neuraxis imaging and persistently negative CSF cytology^5^. Meanwhile, LM is often related with other CNS metastases in the brain parenchyma (33–75%) or dura (16–37%), which may dominate the clinical picture^5^. A comprehensive analysis is required based on the malignant history, clinical manifestation, neuroimaging findings and CSF examinations, in order to achieve the final diagnosis. According to the National Comprehensive Cancer Network (NCCN) guidelines, the diagnostic criteria of LM include CSF positive for tumor cells, positive radiologic findings with supportive clinical findings, signs and symptoms with suggestive CSF (high white blood cell count, low glucose, and high protein) in patients known with a malignancy. NCCN proposes that patients with any of the following criteria are sufficient to the diagnosis: (i) positive CSF cytology; (ii) positive radiologic findings with supportive clinical findings; (iii) LM-related symptoms and signs with abnormal CSF analysis, including high white blood cell count, protein elevation and low glucose in a patient confirmed with malignancy. Among these criteria, there are still disputes in the section (iii)^5^. Furthermore, it was not defined clearly what the positive radiologic findings and the supportive clinical findings were. In some previous studies^4,24^, it was recommended to ascertain the diagnosis by clinical judgments of treating physician. That seems to be subjective and ambiguous. Indeed, it is still lacking of consensus on the diagnostic methods in past LM-related studies.

It is necessary to reveal the clinical characteristics of LM and develop an objective and detailed method for LM diagnosis. Based on the NCCN guidelines, the previous studies^1,4,5,17,24^, the diagnostic criteria and the clinical features revealed in this study, we conceive a quantitative diagnostic method which is coherent and objective, and could be conveniently applied in clinical practice (Table 3). In the criteria, the neuroimaging features and LM-related clinical manifestations are clearly defined. It is necessary to note that the quantitative diagnostic method was also depending on CSF cytology, neuroimaging findings and other clinical findings including malignant tumor history, nervous system symptoms and conventional CSF examination. Therefore, this method could not fundamentally enhance the diagnostic efficiency.

**Table 3:** Quantitative criteria for diagnosis of LM.

Diagnostic factors	Score
**History of malignancy**	0~1
No	0
Yes	1
**Clinical manifestations** ^a^	0~2
None	0
Single symptom in cerebral hemisphere, cranial nerve, or spinal nerves	1
Typical LM-related symptoms or signs^b^	2
**Neuroimaging findings** ^c^	0~4
Normal	0
Suggestive features^d^	1
Diagnostic features^e^	4
**CSF cytology** ^f^	0~5
Negative	0
Suspicious malignant cells	3
Malignant cells	5
**Aberrant CSF biochemical analysis** ^g^	0~1
Normal	0
Abnormal	1

For each patient, a history of malignancy was firstly checked, followed by nuerological examination and neuroimaging examination. Patients with a score of ≥3 should be suggested to receiving CSF examination, and those with a score of ≥5 were confirmed with LM.

After neurological examination, nueroimaging and CSF examination, further examination or review was needed for those with a score of ≤3. Those with a score of 4 were suspicious with LM, and diagnostic treatment for LM (i.e. intrathecal chemotherapy) should be given if the antidiastole (i.e. subarachnoid blood, infection or inflammation) has been excluded. The patients were validated with LM upon remission of the neurological symptoms/signs after diagnostic treatment.

^a^Other conditions that may induced the symptoms should be excluded such as infectious diseases. For patients with symptoms in nerve roots, intervertebral disk degeneration should be excluded. For patients with headache, brain metastasis with obvious space-occupying lesions in MRI or trigeminal neuralgia should be excluded.

^b^Neurological deficits were severe and progressively deteriorative or in multiple sites. Multiple neurological deficits implied disseminated involvement of CNS.

^c^Such features should be confirmed by specialists. Patients simultaneously with diagnostic and suggestive neuroimaging features were bequeathed a score of 4.

^d^Suggestive features included metastatic lesions approaching subarachnoid space, enhancement in dura mater, metastatic lesions approaching ventricles, enhancement in cranial nerves, and communicating hydrocephalus.

^e^Diagnostic features included enhancement in leptomeninges and ependyma, as well as implantation metastases in vertebral canal and ventricles. Any irritation of the leptomeninges (e.g. subarachnoid blood, infection and inflammation) can result in enhancement on MRI. Therefore, these diseases should be excluded.

^f^A volume of ≥10.5 ml was recommended for the CSF sample collection. The samples should be submitted for analysis within 30 minutes.

^g^Aberrant CSF results included elevation of white blood cells and protein(>0.45 g/L), and decrease of glucose(<2.8 mmol/L).

In order to obtain a standardized tool for the evaluation of LM at diagnosis and following treatment, the European Association for NeuroOncology/European Society for Medical Oncology (EANO/ESMO) issued the latest clinical practice guidelines for LM. Just like that stated, they assigned levels of certainty to the diagnosis of LM to provide guidance when to treat (as opposed to when to intensify diagnostic efforts) and on which patients to include in clinical trials. However, with low level of evidence, the recommendations are based mainly on expert opinion and consensus than on evidence collected from clinical trials. The guideline mentioned the LM related clinical presentation, and recommended the criteria of neurological examinations, imaging techniques and CSF exam methods. The guidelines made further efforts to propose diagnostic criteria for LM, also based on neurological symptoms and signs, neuroimaging findings and CSF examinations. The guideline did not fundamentally increase the diagnosis efficiency. In the diagnostic criteria, positive CSF cytology or biopsy is the only basis for the confirmed diagnosis, while the neurological symptoms and signs and neuroimaging findings are the basis for auxiliary diagnosis. The diagnosis is classified into probable, possible or lack of evidence, which contributes to the further management of the disease including LM-directed tumor-specific treatment or follow-up evaluation. This classification provides guidance when to treat with relative confidence (e.g. ‘confirmed’, or ‘probable’) and when to reconsider intensified diagnostic efforts after a firm diagnosis (e.g. ‘possible’, or ‘no evidence for’).

The imaging findings were only classified into linear and nodular types in the EANO/ESMO guidelines. On this basis, the neuroimaging findings are termed as type A (linear), type B (nodular), type C (both linear and nodular) and type D (neither linear or nodular type, e.g. no neuroimaging evidence of LM except possibly hydrocephalus). In the diagnostic criteria, nodular type in the neuroimaging findings with typical clinical signs is defined as probable diagnosis. For treatment, LM-directed tumor-specific treatment is given to the probable diagnosis patients. We think there are still some disputes on it. Firstly, as the guideline mentioned, typical clinical signs of LM include headache, nausea and vomiting, mental changes, gait difficulties, cranial nerve palsies with diplopia, visual disturbances, hearing loss, sensorimotor deficits of extremities and cauda equine syndrome, and radicular neck and back pain. The term “typical clinical signs of LM” is too illusive. For example, these symptoms may present in patients with brain parenchymal metastasis or other neoplastic neurological system disease.

Moreover, there is no clear definition for nodular lesions, and nodular lesions are not specific imaging findings for LM. Nodular lesion with aberrant enhancement is one type of imaging manifestation for brain metastasis. Also, the typical clinical signs of LM mentioned in the guidelines may present secondary to coexistent brain metastases, or treatment-related toxicity. Therefore, in our opinion, nodular lesions with the typical clinical signs of LM (e.g. headache, nausea and vomiting, mental changes, gait difficulties, cranial nerve palsies with diplopia, visual disturbances, hearing loss, sensorimotor deficits of extremities and cauda equine syndrome, and radicular neck and back pain) are not specific for LM. Based on our clinical experiences, the diagnosis of LM is still ambiguous even with the coexistence of these two indictors. We think the patients with probable diagnosis should not be recommended to receive LM-related therapy, such as intrathecal chemotherapy that is considered as an LM-related treatment with risks and toxicity.

In this study, we reviewed and analyzed the LM related imaging findings and clinical presentation retrospectively. In our diagnostic criteria, we classified the LM-related neuroimaging findings and clinical features according to the previous literatures and the patients’ clinical characteristics in this study. We illustrated the imaging findings in a detailed manner. Consulting the previous literature^13^, we classified the imaging characteristics into diagnostic features and suggestive features. Linear type considering as leptomeningeal enhancement was the specific imaging finding for meningitis. LM diagnosis is confirmed in leptomeningeal enhancement cases with a malignant history, and excluding meningitis induced by other conditions such as infectious meningitis or autoimmune meningitis. About the typical clinical signs of LM, the majority of neurological deficits due to LM are commonly irreversible^25^, and these patients present severe and progressively deteriorative. Besides, multiple neurological deficits implied disseminated involvement of CNS. Therefore, the typical LM-related symptoms or signs are defined as severe and progressively deteriorative or multiple neurological deficits in our criteria.

In a word, the EANO/ESMO diagnostic criteria are proposed based on the same diagnostic indicators like the diagnostic criteria and quantitative diagnostic method conceived in this study. The judgment of diagnosis is similar. In our quantitative diagnostic method, we classified the diagnosis into confirmed diagnosis (≥5), suspicious diagnosis (a score of 4), and follow-up evaluation (≤3). Compared with the EANO/ESMO guidelines, we provide detailed illustration and definition of the neuroimaging and clinical features, which is more convenient in clinical practices.

Indeed, there were limitations in this study. Owing to the MRI technical limitations, entire neuraxis MRI can not be performed in our hospital. Moreover, at the initial LM diagnosis, due to severe conditions in many patients, it was quite difficult to perform whole CNS MRI for each patient. Most of the patients merely received neuroimaging examinations for the sites of symptoms/signs. Therefore, we can not present the imaging involvement of these patients exactly. Besides, CSF flow scan was not carried out in our hospital. Thus, the involvement of the entire neuraxis was not well revealed in this study.

## Conclusions

We reviewed a cohort of solid tumor patients diagnosed LM in our hospital over past 6 years. This study provides important information about the clinical features of LM patients with solid tumors.

## Materials and Methods

### Patients

In this retrospective study, we reviewed the clinical data of solid tumors patients with LM admitted in our hospital from August 2009 to December 2015. Data were obtained from LM database which was established since August 2009 to record the clinical information of solid tumor patients diagnosed with LM in our hospital. In the database, LM diagnosis was validated by two experienced neuro-oncologists. The neuroimaging findings and CSF analysis were validated and recorded in the database through a consensus of two neuro-oncologists. Patients with validated primary solid tumors were enrolled. Those with undefined tumors, hematological malignancies, lymphoma and primary CNS tumors (e.g. glioma and intracranial germ-cell tumor) were excluded. Each patient signed the informed consent. The study was performed in line with the Declare of Helsinki, and the protocols were approved by the Ethics Committee of The First Hospital of Jilin University.

### Diagnostic criteria

The diagnostic criteria were established based on the NCCN guidelines and previous literatures^1,4,5,12,17,24^. The LM diagnosis was made in 1 of 5 items (i) malignant cells were detected in CSF; (ii) suspicious malignant cells in CSF, as well as with a malignant history and neurological manifestations; (iii) showing diagnostic neuroimaging features, including leptomeningeal enhancement (any irritation of the leptomeninges, including subarachnoid blood, infection or inflammation should be excluded), subependymal enhancement, or nodules of implantation metastases in vertebral canal or ventricles, consistent with a malignant history or neurological symptoms; (iv) showing typical LM-related neurological symptoms and signs, such as steady headache and nausea, cranial nerves paralysis and spinal nerves symptoms (these neurological deficits were severe and progressively deteriorative or in multiple sites), consistent with suggestive neuroimaging features of LM, including dural enhancement, metastatic lesions approaching subarachnoid space or ventricles, enhancement of cranial nerve, or communicating hydrocephalus, and with a malignant history. Additionally, patients presented abnormal CSF biochemical tests such as protein elevation or decreased glucose levels, excluding a history of traumatic injury of brain or meningitis caused by pathogenic microorganisms; and (v) those with a malignant history and presenting typical LM-related neurological symptoms and signs, while MRI scan was negative or inconsistent with the severe neurological manifestation; as well as excluding other diseases or treatment side effects that may cause these symptoms, and LM-directed treatment (intrathecal chemotherapy) effectively attenuating the existing symptoms.

### Data collection

Clinical information was obtained through reviewing the available records in the LM database of our hospital. The data included date of birth, gender, date of initial diagnosis of primary tumors, site and histology of primary tumors, previous treatment, date of LM diagnosis, KPS score at LM diagnosis, systemic disease status at LM diagnosis, neurological signs and symptoms at LM diagnosis, neuroimaging findings, CSF analysis, LM-related treatment and date of death or last follow-up. The information was reviewed and analyzed by two neuro-oncologists. In some cases, complete information was unavailable as some patients were followed up at outside institutions, either prior to or after LM diagnosis.

### Statistical analysis

A comparison was performed for the clinical features among the patients with different pathological types. Data analysis was performed using SPSS 17.0 software. Chi square test, continuity correction and Fisher’s exact test were used for the comparison of clinical features. Chi square test was used in presence of sample size of ≥40 and a theoretical frequency of ≥5. Continity correction was performed in presence of sample size of ≥40 and a theoretical frequency of less than 5 (≥1). Fisher’s exact test was used in presence of sample size of less than 40 or a theoretical frequency of less than 1. P < 0.05 was considered as statistical difference.

### Consent

This research is strictly retrospective and involving the collection of existing data and records. The study protocol was reviewed and approved consent exemptions by the ethics committee of the First Hospital of Jilin University.

### Novelty and impact

Our study increased the knowledge of LM clinical features. LM patients with different primary tumors showed various clinical characteristics. We hope to raise the recognition of clinical features of LM patients with solid tumors. Such information is beneficial in LM diagnosis.
